# Phase I dose-escalation study of chiauranib, a novel angiogenic, mitotic, and chronic inflammation inhibitor, in patients with advanced solid tumors

**DOI:** 10.1186/s13045-018-0695-0

**Published:** 2019-01-14

**Authors:** Yongkun Sun, Lin Yang, Xuezhi Hao, Yutao Liu, Jinwen Zhang, Zhiqiang Ning, Yuankai Shi

**Affiliations:** 1 Department of Medical Oncology, Beijing Key Laboratory of Clinical Study on Anticancer Molecular Targeted Drugs, National Cancer Center/National Clinical Research Center for Cancer/Cancer Hospital, Chinese Academy of Medical Sciences and Peking Union Medical College, No. 17 Panjiayuan Nanli, Chaoyang District, Beijing, 100021 China; 2 Shenzhen Chipscreen Biosciences Ltd., BIO-Incubator, Suit 2-601, Shenzhen Hi-Tech Industrial Park, Shenzhen, 518057 Guangdong China

**Keywords:** Advanced solid tumors, Chiauranib, Dose-escalation, Pharmacokinetics, Phase I study, Safety

## Abstract

**Background:**

Chiauranib is a novel orally active multi-target inhibitor that simultaneously inhibits the angiogenesis-related kinases (VEGFR2, VEGFR1, VEGFR3, PDGFRα, and c-Kit), mitosis-related kinase Aurora B, and chronic inflammation-related kinase CSF-1R. This phase I dose-escalation study was to determine the maximum tolerated dose (MTD), safety, pharmacokinetics, and preliminary antitumor activity of chiauranib in patients with refractory advanced solid tumor and lymphoma.

**Methods:**

Eighteen patients were treated with continuous dosing of chiauranib from 10 to 65 mg once daily in a dose-escalation 3 + 3 design and evaluated in 28-day cycles. Pharmacokinetic profile of plasma chiauranib was analyzed in both single and multiple dose studies.

**Results:**

Dose-limiting toxicity (DLT) as of grade 3 hypertension occurred in two patients at 65 mg/day, and one dose level below as MTD was 50 mg/day. The most common treatment-related adverse events included fatigue (61.1%), proteinuria (44.4%), hematuria (38.9%), hypothyroidism (38.9%), hypertriglyceridemia (33.3%), and hypertension (33.3%). A linear and dose-dependent pharmacokinetic profile of chiauranib was characterized with rapid absorption and slow elimination feature in both single and multiple dose studies. The accumulative exposure of chiauranib reached the steady state within 8 days and was approximately increased by twofold as those in the single dose study. No complete or partial response was observed, and 12 patients (66.7%) achieved stable disease (SD).

**Conclusions:**

Chiauranib demonstrated an acceptable safety and favorable pharmacokinetic profile with potential antitumor activity. Several phase Ib/II clinical studies are currently under further investigation.

**Trial registration:**

NCT, NCT02122809. Registered 25 April 2014

## Background

Tumorigenesis is a multi-step process involving in distinctive and complementary hallmark capabilities that enable tumor growth and metastatic dissemination [1]. Accordingly, the phenotypic and functional heterogeneity of tumor cells especially in tumor microenvironment even add another dimension into the complexity of tumor expansion [2]. Therefore, instead of single-target inhibitors currently used in cancer therapy, multi-targeted tyrosine kinase inhibitors (TKIs) that influence multiple key signal pathways in different type of tumor cells could overcome the limitations from single-target inhibition and be more efficient as promising therapeutic drugs due to their unique theoretical bases and anti-tumor mechanisms [3, 4].

Chiauranib is a novel orally active multi-target inhibitor that simultaneously inhibits three major pathways in tumorigenesis, including the angiogenesis-related kinases (VEGFR2, VEGFR1, VEGFR3, PDGFRα, and c-Kit), mitosis-related kinase Aurora B, and chronic inflammation-related kinase CSF-1R. The compound shows very high selectivity and potency in the inhibition of these kinases with the IC50 at a single-digit nanomolar range, but with little activity on off-target non-receptor kinases, proteins, GPCR, and ion channels [5]. Target inhibition and effects on the corresponding biological pathways of chiauranib have been verified in vivo by preclinical studies. Also, chiauranib shows broad activity against human tumor xenograft models inoculated with human cancer cell lines derived from colon, lung, liver, or stomach cancers [5].

Based on the promising preclinical antitumor activity, this phase I study was designed to determine the safety and tolerability of chiauranib in patients with advanced solid tumor or lymphoma. The pharmacokinetic profile and preliminary antitumor activity were also evaluated as secondary objectives.

## Methods

### Chemicals and reagents

Chiauranib capsules were provided by Shenzhen Chipscreen Biosciences, Ltd. (Shenzhen, China). Methanol and acetonitrile were of HPLC grade (Fisher Scientific, Fair Lawn, NJ, USA). All other chemicals were of analytical reagent grade. Distilled, deionized water was produced by a Milli-Q Reagent Water System (Millipore, MA, USA).

### Eligibility criteria

Eligible patients had histological or cytological confirmation of advanced solid tumor, or non-Hodgkin lymphoma (NHL), refractory to or without standard therapy options. Other inclusion criteria were as follows: body mass index (BMI) was between 18 and 28; age was between 18 and 65; Eastern Cooperative Oncology Group (ECOG) performance status was of 0 to 1; acceptable liver, renal, and bone marrow function including normal serum electrolytes were mandatory: hemoglobin (Hb) ≥ 100g/L (no blood transfusion within 14 days); absolute neutrophil count (ANC) ≥ 1.5 × 10^9^/L; platelets ≥ 100 × 10^9^/L; serum creatinine ≤ 1.5× upper limit of normal (ULN); total bilirubin ≤ 1.5× ULN; alanine aminotransferase (ALT)/aspartate aminotransferase (AST) ≤ 1.5× ULN; fasting triglyceride (TG) ≤ 3.0 mmol/L; total cholesterol ≤ 7.75 mmol/L; and International Normalized Ratio (INR) < 1.5. Women of childbearing potential should be non-lactating patients and must agree to use effective contraceptive methods prior to study entry, during study participation, and up to 6 months following completion of therapy. A serum or urine pregnancy test within 7 days before enrollment must be negative. Men must agree to use effective contraceptive methods during study participation and up to 6 months following completion of therapy.

Major exclusion criteria included patients with life expectancy less than 3 months, or with uncontrolled or significant cardiovascular diseases, or with any anti-cancer treatment within 4 weeks prior to study entry. Other exclusion criteria were as follows: active bleeding, or current thrombotic disease, or using anticoagulants; history of deep venous thrombosis or pulmonary embolism; clinical significant gastrointestinal abnormality; brain metastasis with symptoms; and organ transplant recipients, proteinuria positive, congenital or acquired immunodeficiency, active infection, and mental disorders were ineligible.

The study (ClinicalTrials.gov ID: NCT02122809) was conducted according to good clinical practice guidelines and the Declaration of Helsinki. The Institutional Review Boards of the Cancer Hospital, Chinese Academy of Medical Sciences and Peking Union Medical College, approved the study, and written informed consent was obtained from all patients prior to performing study-related procedures.

### Study design

This was an open-label, dose-escalation (3 + 3 design) cohort study of single-agent chiauranib. The primary objective of the study was to determine dose-limiting toxicity (DLT), maximum tolerated dose (MTD), and safety profile. Secondary objectives were to characterize the pharmacokinetic profile and preliminary antitumor activity of chiauranib.

### Drug administration

Chiauranib was supplied by Shenzhen Chipscreen Biosciences, Ltd. In order to evaluate the initial pharmacokinetics (PK) parameters after one dose, a 6-day-lead-in period was designed in the first treatment cycle, followed by the daily administration of chiauranib capsules. A treatment cycle was defined as 28 days. Chiauranib capsules were administered with fasting condition.

Based on the overall results from preclinical studies and the principle for human equivalent dose (HED) analysis, 25 mg/day would be suggested as the starting dose for chiauranib in human. The executed starting dose in the study was 10 mg/day, considering a safety factor of 2.5, and only two patients were required to enroll in this group. The escalating doses were 20 mg, 35 mg, 50 mg, and 65 mg, determined by a modified Fibonacci sequence until the MTD was achieved.

### Dose-escalation procedures

Patients were enrolled sequentially into dose escalation cohorts using a standard 3 + 3 design, except that only two patients were enrolled at the starting dose. Intra-patient dose escalation was not permitted.

If one of the first three patients at a dose level experienced DLT, up to three additional patients (total up to six patients) were enrolled at that dose level. If more than two patients at a dose level experienced DLT, dose escalation was halted, and the dose level was determined to have exceeded MTD. Three additional patients were then entered on the next lower dose level. MTD was defined as the highest dose with an observed incidence of DLT in no more than one of six patients.

### DLT definitions

Toxicity was graded according to Common Terminology Criteria for Adverse Events (CTCAE) version 4.0. DLT was defined as any grade ≥ 2 nephrotoxicity, or any grade ≥ 3 non-hematologic toxicity, or grade ≥ 4 hematologic toxicity occurring during the first cycle at least possibly related or of unknown relationship to chiauranib.

### Safety assessment and clinical evaluation

Safety assessments included the reporting of all adverse events (AEs), changes in clinical laboratory parameters (hematology, serum chemistries, coagulation, urinalysis), vital sign measurements, 12-lead electrocardiogram (ECG) results, and physical examination findings. In addition, serial measurements of left ventricular ejection fraction, thyroid function, myocardial enzymes, troponin, amylase, and lipase were performed. Twenty-four-hour urine protein was implemented if clinically indicated.

Safety parameters were assessed at baseline and at regular intervals during the study. Adverse events (AEs) were classified/graded weekly according to the CTCAE version 4.0.

Tumor response was evaluated by the Response Evaluation Criteria in Solid Tumors (RECIST) version 1.1. Tumor response was assessed by computed tomography (CT) and/or magnetic resonance imaging (MRI) scans at screening and every 8 weeks (2 cycles) during the first 12 cycles. And the investigator could adjust the assessing intervals according to specific conditions of the patient thereafter, if patients continued to receive treatment with chiauranib for 13 or more cycles.

### Pharmacokinetic sampling and analysis

In the initial PK evaluation after one dose, the eligible patients received a single oral dose of 10, 20, 35, 50, and 65 mg chiauranib capsules under fasting condition. Three milliliters of venous blood samples was drawn into heparinized tubes before drug administration as well as at 1, 2, 4, 6, 8, 12, 24, 48, 72, 96, 120, and 144 h post-dose. In the multiple dose study, patients continuously received from 10 to 65 mg chiauranib capsules once daily at the first treatment cycle. Three milliliters of venous blood samples was drawn into heparinized tubes before administration as well as at 1, 2, 4, 8, 12, and 24 h post the oral dose on day 28. To evaluate the steady-state trough concentration of chiauranib, blood samples were also collected before dosing on days 8, 15, 22, 25, 26, and 27 of the first cycle, and on each day 28 from the second cycle until the end. Samples were centrifuged immediately at 3600×*g* for 10 min at 4 °C, and the plasma fractions were separated and stored at − 20 °C until analysis.

The plasma concentration of chiauranib was determined by a validated LC-MS/MS method with lower limits of quantification (LOQ) at 1.00 ng/mL. The intra- and inter-day precisions and accuracies (relative standard deviation, %RSD) were in the range of 1.75–6.33%, and − 7.88–9.00%, respectively. The extraction recovery (relative standard deviation, %RSD) was 1.32–5.16%.

### Statistical methods

Descriptive statistics were used for baseline characteristics, safety assessments, pharmacokinetic variables (including *C*_max_, *T*_max_, AUC, and *t*_1/2_) and exploratory efficacy assessments, including tumor response and time to progression. Continuous variables were summarized with means, standard deviations (SD), medians, minimums, and maximums. Categorical variables were summarized by counts and by percentage of patients in corresponding categories. *P* values < 0.05 were considered statistically significant. Statistical analyses were performed using SAS® v9.4 (Cary, NC).

## Results

### Patient characteristics

Eighteen patients (6 female, 12 male) were enrolled from March 7, 2014, to December 30, 2015. The median age was 49.5 years (range 23–65). Eight patients (44%) were of ECOG PS 0. All patients had confirmed progressive metastatic diseases at study entry with a median of 3.5 prior regimens of systemic therapies (range 1–9). The most common primary malignancies were colorectal cancer (7 cases, 38.9%), non-small cell lung cancer (NSCLC) (5 cases, 27.8%), and others (6 cases) with multiple primary cancers. Baseline patient and tumor characteristics are detailed in Table 1.

**Table 1:** Baseline demographics and patient characteristics

Characteristic	Total (*N* = 18)	
Sex, *n* (%)		
Male	12 (66.7)	
Female	6 (33.3)	
Age (mean), years	49.1	
Median (range)	49.5 (23–65)	
ECOG performance status, *n* (%)		
0	8 (44.4)	
1	10 (55.6)	
TNM stage, *n* (%)		
IIIA	1 (6)	
IV	17 (94)	
Previous regimens of systemic therapies		
Median (range)	3.5 (1–9)	
Tumor type, *n* (%)		
NSCLC	5 (27.8)	
Colorectal cancer	7 (38.9)	
Ovarian cancer	1 (5.6)	
Gastric cancer	1 (5.6)	
Thyroid cancer	1 (5.6)	
DLBCL	1 (5.6)	
Fibrosarcoma	1 (5.6)	
Renal cancer	1 (5.6)	

*ECOG* Eastern Cooperative Oncology Group, *NSCLC* non-small-cell lung cancer, *DLBCL* diffuse large B cell lymphoma

### Dose exposure and modifications

A total of 87 treatment cycles were administered to 18 patients at 5 different dose levels during the study. The duration of treatment was consistent across the dose levels, with a median of 3 cycles being administered at all dose levels (range 1–23). Ten patients (55.6%) were continued for treatment after 2 cycles, and all of them completed at least 3 cycles with the longest duration of treatment for 23 cycles. At the time of data analysis, all 18 patients stopped therapy for following reasons: 14 (77.8%) due to disease progression, 1 (5.6%) due to DLT (treatment-related grade 3 non-hematologic toxicity) at dose 65 mg during cycle 1, 1 (5.6%) due to withdrawal of consent, and 2 (11.1%) due to investigator’s discretion. For the investigator’s discretion of treatment termination, 1 patient was due to poor general condition in her late-stage disease. The other patient with diffuse large B cell lymphoma was treated by the study drug at 10 mg for 23 cycles with long-lasting stabilized disease lesions showing no metabolism activity by Positron emission tomography-computer tomography (PET-CT) assessment.

Of the 18 enrolled patients, none underwent dose modifications or reductions, but treatment delay was implemented on 2 (11.1%) patients because of adverse events: 1 patient in the 10 mg cohort omitted doses for 5 days due to grade 1 nasopharyngitis and 1 patient treated at dose level 50 mg omitted treatment for 10 days due to grade 1 elevation of AST and ALT. These adverse events resolved fully following brief drug holidays. The elevation of AST and ALT was considered by the investigator to be possibly related to chiauranib, and the nasopharyngitis was considered to be unlikely related. All patients who received study treatment were evaluable for safety, pharmacokinetics, and efficacy.

### Dose escalation and recommended phase II dose

All the 18 patients were evaluable for the determination of MTD during the first 4 weeks of treatment only (1 cycle). Two patients were enrolled in the initial dosing cohort of 10 mg daily. Dose levels were escalated in four additional cohorts: 20 mg daily (*n* = 6), 35 mg daily (*n* = 3), 50 mg daily (*n* = 3), and 65 mg daily (*n* = 4). Two of the 4 patients at the highest dose level experienced protocol-defined DLTs of grade 3 hypertension during cycle 1, prompting a decision not to escalate further. Hypertension only resulted in treatment discontinuation for 1 patient, while the other patient who underwent DLT was assessed by the investigator to continue treatment due to potential and promising benefit from chiauranib. Except for hypertension, no other DLT was reported during the study (All other events were either not considered to be related to treatment by the investigator or did not conform to the DLT definition).

Based on the safety data, 50 mg chiauranib administered orally once daily was considered to be both the MTD and the RP2D.

### Safety and adverse event profile

Chiauranib was generally well tolerated, and the majority of AEs were mild to moderate in severity. No treatment-related deaths occurred during the study. All 18 patients experienced at least one AE, and 17 patients (94.4%) reported at least one treatment-related AE. The most common treatment-related AEs, irrespective of grade, were fatigue (61.1%), proteinuria (44.4%), hematuria (38.9%), hypothyroidism (38.9%), hypertriglyceridemia (33.3%), and hypertension (33.3%). There were no obvious abnormal laboratory parameters for renal function observed, such as serum urea nitrogen and creatinine. Table 2 summarized the most common treatment-related AEs occurring in at least 10% of patients. All these treatment-related AEs were of grade 1 or 2 severity with the exception of five cases, which were grade 3 hypertension (in two patients), neutropenia (in two patients), and hyperglycemia (in one patient). No treatment-related AE exceeded grade 3 was observed.

**Table 2:** Treatment-related adverse events: worst grade per patient in all cycles in at least 10% of all patients, *n* (%)

Adverse event	Grade^a^					Total
	1	2	3	4	5	
Blood and lymphatic system disorders						
Hypertension	3 (16.7)	1 (5.6)	2 (11.1)	0	0	6 (33.3)
Vaginal hemorrhage	2 (11.1)	0	0	0	0	2 (11.1)
Endocrine disorders						
Hypothyroidism	7 (38.9)	0	0	0	0	7 (38.9)
Metabolism and nutrition disorders						
Hypertriglyceridemia	5 (27.8)	1 (5.6)	0	0	0	6 (33.3)
Decreased appetite	0	2 (11.1)	0	0	0	2 (11.1)
Respiratory, thoracic, and mediastinal disorders						
Dysphonia	2 (11.1)	0	0	0	0	2 (11.1)
Gastrointestinal disorders						
Nausea	4 (22.2)	0	0	0	0	4 (22.2)
Abdominal pain	2 (11.1)	0	0	0	0	2 (11.1)
Diarrhea	2 (11.1)	1 (5.6)	0	0	0	3 (16.7)
Mouth ulceration	1 (5.6)	1 (5.6)	0	0	0	2 (11.1)
Renal and urinary disorders						
Proteinuria	8 (44.4)	0	0	0	0	8 (44.4)
Hematuria	7 (38.9)	0	0	0	0	7 (38.9)
Skin and subcutaneous tissue disorders						
Rash	1 (5.6)	1 (5.6)	0	0	0	2 (11.1)
Palmar-plantar erythrodysesthesia syndrome	1 (5.6)	1 (5.6)	0	0	0	2 (11.1)
General disorders and administration site conditions						
Fatigue	7 (38.9)	4 (22.2)	0	0	0	11 (61.1)
Lab examinations						
Leucopenia	2 (11.1)	1 (5.6)	0	0	0	3 (16.7)
ALT increased	2 (11.1)	0	0	0	0	2 (11.1)
Amylase increased	3 (16.7)	1 (5.6)	0	0	0	4 (22.2)
Conjugated bilirubin increased	5 (27.8)	0	0	0	0	5 (27.8)
Neutropenia	2 (11.1)	0	2 (11.1)	0	0	4 (22.2)
AST increased	3 (16.7)	0	0	0	0	3 (16.7)
Hyperbilirubinemia	2 (11.1)	0	0	0	0	2 (11.1)
Unconjugated bilirubin increased	2 (11.1)	0	0	0	0	2 (11.1)
Blood urea increased	2 (11.1)	0	0	0	0	2 (11.1)
Thrombocytopenia	2 (11.1)	0	0	0	0	2 (11.1)

Patients with multiple events in the same category are counted only once in that category; patients with events in more than one category are counted once in each of those categories

*ALT* alanine amino-transferase, *AST* aspartate amino-transferase

^a^Graded per National Cancer Institute Common Terminology Criteria for Adverse Events, version 4.0

### Serious adverse event

A total of one serious adverse event (SAE) was reported. One patient with advanced NSCLC treated at the 20-mg dose level experienced AEs comprising grade 2 cough, fatigue, and anorexia during the first cycle, and the investigator made a decision that the patient withdraw from the study due to her poor condition. This patient died from deteriorating pulmonary infection 15 days after treatment termination of the study drug. Although this death was assessed as unlikely related to the study drug, six patients were enrolled in the 20 mg cohort in this study.

### Pharmacokinetic studies

**Fig. 1:**
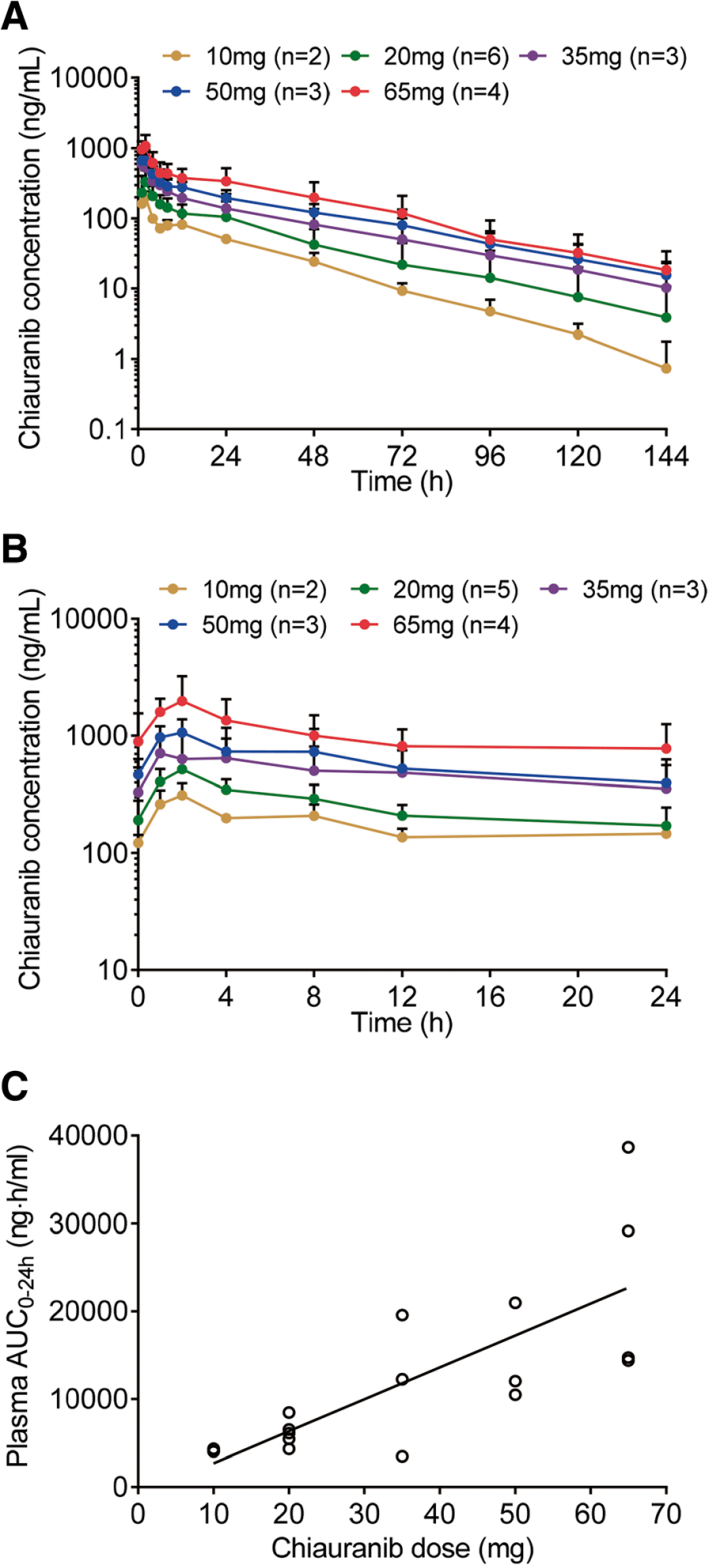
Pharmacokinetic analyses of chiauranib. **a** The mean plasma concentration-time curves of chiauranib in patients at a single-oral dose of 10, 20, 35, 50, and 65 mg chiauranib. The mean plasma concentration-time curves (**b**) and AUC-dose linear regression (**c**) of chiauranib on day 28 in the first treatment cycle at a multiple oral dose of 10, 20, 35, 50, and 65 mg chiauranib

Over the dose escalation from 10 to 65 mg in patients, chiauranib plasma exposures showed a dose proportional and good linear pharmacokinetic profile in both single and multiple dose studies (Fig. 1a–c). After the first dose administration in run-in period, chiauranib exhibited a rapid absorption with a median peak time of 2 h (range from 1 to 4 h), and a relatively slow elimination with the terminal half-life (*t*_1/2_) of 25.6 ± 6.73 h. The accumulative exposure of chiauranib such as mean AUC and *C*_max_ on day 28 in the first treatment cycle was dose proportional and approximately increased by twofold compared to those in the single dose study. Notably, the continuous dosing of chiauranib did not result in the saturable absorption even at the clinical DLT of 65 mg. The plasma exposure parameters of chiauranib for both single and multiple dose are listed in Table 3.

**Table 3:** The pharmacokinetic exposure parameters of plasma chiauranib after single and multiple dose of 10, 20, 35, 50, and 65 mg chiauranib capsules in patients with advanced solid tumor

		*T*_max_ (h)	AUC_0–24 h_ (ng h/mL)	AUC_0–144 h_ (ng h/mL)	*C*_max_ (ng/mL)	DF	*C*_trough_ (ng/mL)	*C*_av_ (ng/mL)
Day1	10 mg (*n* = 2)	2.0	1998	3576	201	/	/	/
	20 mg (*n* = 6)	2.0 (1–4)	3454 (1275)	6815 (3902)	354 (158)	/	/	/
	35 mg (*n* = 3)	1.0 (1–8)	5762 (3853)	11859 (8656)	608 (437)	/	/	/
	50 mg (*n* = 3)	1.0 (1–2)	7417 (1380)	16429 (5251)	756 (87)	/	/	/
	65 mg (*n* = 4)	1.5 (1–2)	11015 (3572)	24823 (12581)	1275 (358)	/	/	/
Day28	10 mg (*n* = 2)	2.0	4185	/	312	1.1 (0.4)	122 (21)	174 (9)
	20 mg (*n* = 6)	2.0	6175 (1526)	/	517 (134)	1.3 (0.6)	191 (89)	257 (64)
	35 mg (*n* = 3)	4 (1–4)	11761 (8066)	/	773 (532)	0.8 (0.4)	330 (208)	490 (336)
	50 mg (*n* = 3)	2 (1–2)	14504 (5650)	/	1118 (254)	1.1 (0.3)	467 (166)	604 (235)
	65 mg (*n* = 4)	2 (1–2)	24230 (11819)	/	2238 (1012)	1.5 (0.8)	890 (662)	1010 (492)

*AUC*_*0–24 h*_ area under the concentration-time curve from 0 to 24 h, *AUC*_*0–144 h*_ area under the concentration-time curve from 0 to 144 h, *C*_*max*_ maximum plasma concentration, *T*_*max*_ time to reach maximum plasma concentration

**Fig. 2:**
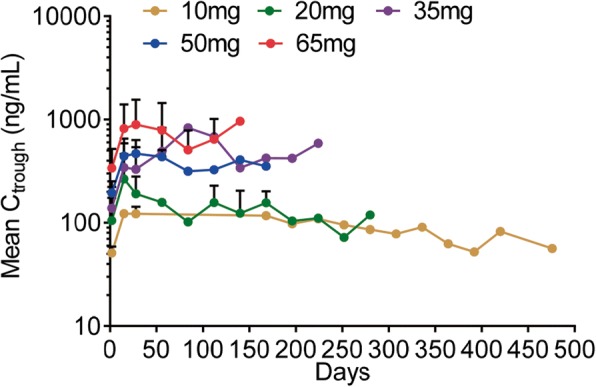
Steady-state mean trough concentration (*C*_trough_) of chiauranib. Mean *C*_trough_ after a continuous oral dose of 10, 20, 35, 50, and 65 mg chiauranib capsules once daily in patients throughout the treatment cycles were shown

The plasma exposure of chiauranib reached the steady state from day 8 upon continuous once daily administration. The trough plasma concentration of chiauranib was consistently stable from the first treatment cycle, and increased by twofold compared to that after the first administration of chiauranib (Fig. 2).

### Anti-tumor activity

**Fig. 3:**
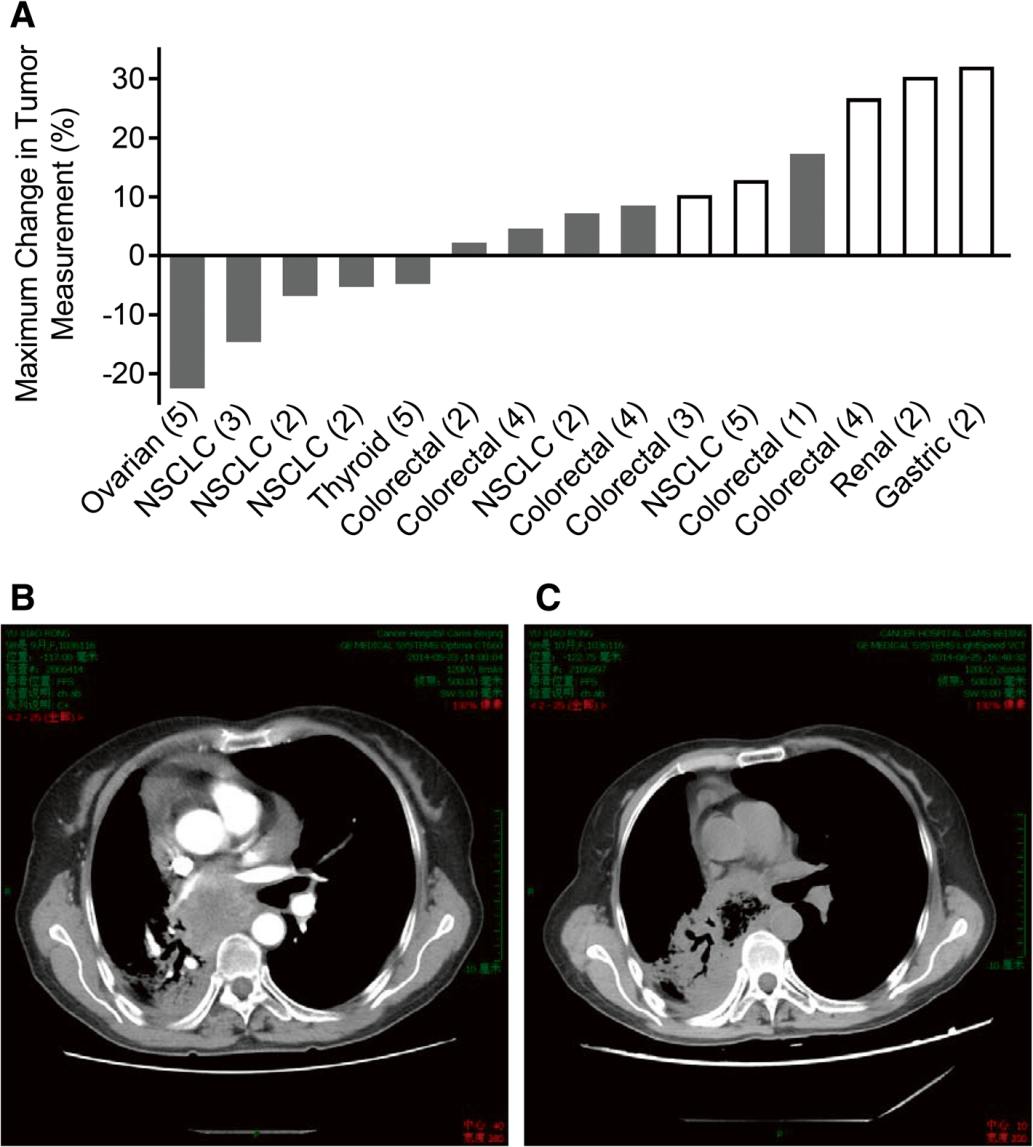
Waterfall plot of changes in the target lesions with a representative case of clinical response. **a** Chiauranib dose cohorts are shown in parentheses as 1 = 10 mg, 2 = 20 mg, 3 = 35 mg, 4 = 50 mg, and 5 = 65 mg. Open bars represent patients who had progressive disease, and the filled bars represent patients who achieved stable disease as their best responses. Not shown, *n* = 3, due to unavailable tumor measurements. Coronal chest CT at baseline (**b**) and after 4 weeks chiauranib treatment (**c**) from a 59-year-old woman with poorly differentiated adenocarcinoma of the lung, who has been received surgery, adjuvant chemotherapy, chemo-radiation, and two salvage chemotherapies

Tumor response was assessed during the study. Among the 18 patients enrolled, 15 had baseline measurable diseases evaluable for tumor response by RECIST 1.1. No partial or complete response was observed; 12 patients (66.7%) had stable disease (SD), and 6 patients (33.3%) had progressive disease (PD) as best response to therapy based on the investigator’s assessment. Figure 3a represents the changes in the sum of the longest diameters of target lesions observed at the time of best response.

Patients who experienced clinical benefit for greater than 8 weeks included two patients with colorectal cancer (one patient each in the 20 and 50 mg cohorts), two patients with NSCLC (one patient each in the 20 and 35 mg cohorts), one patient with ovarian cancer (65 mg cohort), and one patient with DLBCL (10 mg cohort). Typically, one patient with NSCLC (poorly differentiated adenocarcinoma) in the dose of 20 mg cohort had showed diminished pericardial effusion and tumor shrinkage with a cavity in the chest CT at the time of 4 weeks treatment (Fig. 3b, c).

## Discussion

With the advanced molecular biology and understanding of tumorigenesis, an increasing number of kinase-mediated signaling pathways have been identified as potential therapeutic targets for the treatment of hematologic malignancies and solid tumors [6]. Chiauranib is a novel orally active multi-target inhibitor that simultaneously inhibits the angiogenesis-related kinases (VEGFR2, VEGFR1, VEGFR3, PDGFRα, and c-Kit), mitosis-related kinase Aurora B, and chronic inflammation-related kinase CSF-1R. The potential anti-tumor activities of chiauranib have been previously demonstrated both in vitro and in vivo xenograft tumor models [5].

The current study was undertaken to determine the safety, DLT, and RP2D of chiauranib. In general, chiauranib was well tolerated with mild to moderate toxicity profile, and the most frequently reported treatment-related AEs were fatigue, proteinuria, hematuria, hypothyroidism, hypertriglyceridemia, hypertension, bilirubin increased, and neutropenia, as expected from the mechanism of action of the drug. For example, proteinuria and hypothyroidism have also been reported with other VEGFR TKIs, probably due to a loss of endothelial fenestrations and podocytes [7] and thyroid atrophy from reduction of vessel density [8]. Neutropenia is another common toxicity in our study, which could be related to the inhibition of Aurora B [9] and CSF-1R kinases [10].

During dose escalation, two cases of grade 3 hypertension were observed as DLTs at 65 mg cohort. Hypertension is often a dose-limiting toxicity for VEGFR inhibition, which may be a mechanism-dependent, “on-target” toxicity of the VEGF signaling pathway rather than non-specific effects on unrelated signaling pathways [11, 12]. Other DLTs of VEGFR inhibitors including fatigue, skin rash, palmar-plantar erythrodysesthesia syndrome, and diarrhea were observed in early clinical studies [7, 13, 14]. DLTs in phase I trials of Aurora kinase inhibitors were primarily hematological events, particularly neutropenia [15–18], and DLTs for CSF-1R inhibitor included INR increase, lymphopenia, AST increase, anemia, neutropenia, and syncope [10]. As for multi-target inhibitors, Ilorasertib (ABT-348) targeting Aurora kinase A/B, VEGFR/PDGFR and Src family, and ENMD-2076 targeting Aurora kinase A, VEGFR2/FGFR/Flt-3/c-Kit, were found to cause more DLTs, such as pancreatic disease and hypertension, fatigue, cholecystitis, and QTc prolonged [19, 20]. Comparing to these single- or multi-target inhibitors above mentioned, chiauranib showed less DLTs and all the grade 3 AEs such as hypertension, hyperglycemia, and neutropenia were highly manageable. On the basis of these results, the PR2D of chiauranib was 50 mg for once-daily administration as monotherapy.

Chiauranib was well absorbed and showed favorable pharmacokinetic parameters. Chiauranib performed typical linear pharmacokinetic profile in both single and multiple dose studies, in which the main parameters *C*_max_ and AUC showed linear relation to the dosages. And it did not present any evidence of the saturation of absorption even at the dose of DLT. Compared to the drug exposure after the single-dose administration, the accumulative exposure of chiauranib in the steady state was approximately increased by twofold. Also, the steady state *C*_trough_ was consistent and relatively stable in patients from the first treatment cycle. Overall, the results of single/multiple doses study indicate that chiauranib has well pharmacokinetic characters with tolerance and safety, which provide useful information for the further clinical trial.

In the present phase 1 trial, 12 patients (66.7%) achieved SD as the best response. No complete or partial responses were observed. All of these cases who achieved SD greater than 8 weeks included colorectal cancer, NSCLC, ovarian cancer, and DLBCL. Among them, one patient with ovarian cancer demonstrated a marginal partial response with 22.2% change in the sum of the longest diameters of target lesions observed at the time of best response. Furthermore, one patient with DLBCL in the 10 mg cohort experienced a confirmed SD lasting for 596 days.

Notably, in this heavily pretreated population, six patients (6/18, 33.3%) with a variety of solid tumors, having received prior anti-angiogenic agents, also showed prolonged clinical benefits. While five patients achieved SD, four among them remained on chiauranib for at least 16 weeks. This may indicate a better clinical efficacy of chiauranib compared with other anti-angiogenic agents, likely because of the concomitant inhibition of Aurora kinase and CSF-1/CSF-1R signaling pathways. Possibly, prior exposure to antiangiogenic therapy did not preclude clinical benefit of chiauranib. Although a correlation between the development of hypertension and clinical benefit has been reported for a number of antiangiogenic agents, including bevacizumab, sunitinib, and sorafenib [21–24], we did not observe this association with chiauranib.

In summary, this first-in-human study demonstrated that the novel, angiogenic, Aurora, and CSF1-R kinase inhibitor chiauranib is well tolerated and can be safely administered to cancer patients with a PR2D of 50 mg QD once daily. Favorable PK properties were confirmed, and signs of clinical activity were elicited. Evaluation of chiauranib as monotherapy in four phase Ib/II clinical trials of relapsed/refractory ovarian cancer, NHL, hepatic cell carcinoma, and small cell lung cancer patients after standard systemic therapy (ClinicalTrials.gov NCT03166891, 03074825, 03245190, and 03216343) are currently undergoing.

## Abbreviations

AE: Adverse event
CI: Confidence interval
*C*_max_: Maximum plasma concentration
CTCAE: National Cancer Institute Common Terminology Criteria for Adverse Events
CYP: Cytochrome
DLBCL: Diffuse large B cell lymphoma
DLT: Dose-limiting toxicity
ECOG: Eastern Cooperative Oncology Group
MTD: Maximum tolerated dose
NA: Not applicable
NSCLC: Non-small cell lung cancer
PK: Pharmacokinetics
RECIST: Response Evaluation Criteria in Solid Tumors
RP2D: Recommended phase II dose
SAE: Serious adverse event
SD: Stable disease
TKI: Tyrosine kinase inhibitor
*T*_max_: Time to reach maximum plasma concentration
WHO: World Health Organization
